# Stereocomplexed microparticles loaded with *Salvia cadmica* Boiss. extracts for enhancement of immune response towards *Helicobacter pylori*

**DOI:** 10.1038/s41598-023-34321-6

**Published:** 2023-04-29

**Authors:** Weronika Gonciarz, Magdalena Chmiela, Bartłomiej Kost, Ewelina Piątczak, Marek Brzeziński

**Affiliations:** 1 Department of Immunology and Infectious Biology, Faculty of Biology and Environmental Protection, Institute of Microbiology, Biotechnology and Immunology, University of Lodz, Banacha 12/16, 90-237 Lodz, Poland; 2 Centre of Molecular and Macromolecular Studies, Polish Academy of Sciences, Sienkiewicza 112, 90-636 Lodz, Poland; 3 Department of Pharmaceutical Biotechnology, Medical University of Lodz, Muszyńskiego 1, 90-151 Lodz, Poland

**Keywords:** Drug delivery, Gastrointestinal diseases

## Abstract

Controlled delivery of therapeutic substance gives numerous advantages (prevents degradation, improves uptake, sustains concentration, lowers side effects). To encapsulate *Salvia cadmica* extracts (root or aerial part), enriched with polyphenols with immunomodulatory activity, in stereocomplexed microparticles (sc-PLA), for using them to enhance the immune response towards gastric pathogen *Helicobacter pylori*. Microparticles were made of biodegradable poly(lactic acid) (PLA) and poly(d-lactic acid) (PDLA). Their stereocomplexation was used to form microspheres and enhance the stability of the obtained particles in acidic/basic pH. The release of *Salvia cadmica* extracts was done in different pH (5.5, 7.4 and 8.0). The obtained polymers are safe in vitro and in vivo (guinea pig model). The sc-PLA microparticles release of *S. cadmica* extracts in pH 5.5, 7.4, and 8.0. *S. cadmica* extracts enhanced the phagocytic activity of guinea pig bone marrow-derived macrophages, which was diminished by *H. pylori*, and neutralized *H. pylori* driven enhanced production of tumor necrosis factor (TNF)-α and interleukin (IL)-10. The sc-PLA encapsulated *S. cadmica* extracts can be recommended for further in vivo study in guinea pigs infected with *H. pylori* to confirm their ability to improve an immune response towards this pathogen.

## Introduction

Controlled delivery of biologically active therapeutic substances gives numerous advantages, as it reduces premature drug degradation, improves drug uptake and sustains its’ concentrations within the therapeutic window, and lowers side effects. In recent years, active substances have been delivered to the host using different carriers, due to their large volume-to-surface area ratio, modifiable external shell, biodegradability, biocompatibility, low cytotoxicity, as well as targeting and stimulus-responsive capabilities^1^. Most drug carriers are made of biodegradable poly(lactic acid) (PLA) and poly(lactic-co-glycolic acid) (PLGA) matrices^2^, which are biocompatible and low immunogenic by themselves^3,4^. However, their resistance to acidic^5^ and basic conditions^6^ is limited. This specific type of physical cross-linking of these materials to enhance their hydrolytic degradation in such conditions has been suggested. One strategy relies on the formation of stereocomplexes^7^. These complexes are formed by multiple H-bonds between poly(l-lactide) (PLLA) and poly(d-lactide) (PDLA) chains. The formation of such novel crystalline structure leads to the enhanced hydrolytic resistance^8^. The increase in crystallinity causes a slower rate of degradation because semicrystalline or crystalline polymers start the degradation in the amorphous parts and continue in the crystalline ones when all the amorphous regions are degraded^9^. Moreover, stereocomplexation methodology is used for the preparation of microparticles by spontaneous precipitation from organic solvents^10,11^. It has been shown that stereocomplex microparticles can be considered resistant to acids and bases only at certain mild conditions (pH 1.0 or pH 13.0) and really short time (to 30 min)^12^, and due to this they can be used as carriers delivering an active substances.

Gram-negative rods *Helicobacter pylori* colonize gastric or duodenal mucosa in humans (prevalence 50%-90%) where they are involved in the development of acute and then chronic gastritis or gastroduodenitis, gastric ulcers, and even gastric cancer^13,14^. They destroy the gastric barrier due to the induction of oxidative stress and apoptosis^15–18^, and diminish the activity of immune cells of the host including phagocytes^19–21^, NK cells^22^, or T lymphocytes^23^. This may result in the maintenance of infection. Currently, high rate resistance of *H. pylori* to antibiotics (including amoxicillin, clarithromycin, metronidazole, and levofloxacin) becomes a problem^24–26^, and requires finding new therapeutic substances released in an appropriate pH, in a concentration showing the desired biological activity.

In the previous study, we showed that *Salvia cadmica* root (SCRE) or aerial part (SCAPE) hydromethanolic extracts, which are enriched with polyphenols, poses anti-*H. pylori* activity, neutralize the deleterious effects of oxidative stress driven by *H. pylori* lipopolysaccharide (LPS), and promote regeneration of gastric epithelial cells and fibroblasts in vitro^27^. These extracts have been evaluated as non-cytotoxic thus, they can undergo a further study to develop a therapeutic formulation. Plant extracts enriched with polyphenols, which could be delivered to the gut are good candidates for priming immunocompetent cells^28–30^.

In the present study, the encapsulation *S. cadmica* extract in stereocomplexed PLA microparticles (sc-PLA), to enhance the immune response towards *H. pylori* was performed.

We plan to encapsulate *S. cadmica* extracts in sc-PLA in order to protect them from acidic pH in the stomach. The proposed insertion strategy is due to oral administration of such sc-PLA with encapsulated extracts. Stability of PLA-like particles in acidic environment can be improved by transformation them into supramolecular complexes–stereocomplexes, due to blending two enantiomeric forms of PLA^7^. The carrier in form resistant to acidic milieu will help to deliver the unchanged plant extract to the gut where the released biocomponents will show their activity towards immune cells. Previously we showed using a THP-1 macrophage model, that *S. cadmica* extracts preserve the phagocytic activity of macrophages, which was lost in cells primed with *H. pylori* or these bacilli LPS. The enhancement of macrophage phagocytic activity was associated with deposition of surface adhesins CD11b and CD11d and downregulation of *H. pylori* LPS induced pro-inflammatory cytokines. These effects driven by *S. cadmica* extracts were related to the modulation of the NF-κB signaling pathway^31^. Furthermore we selected sc-PLA for the study due to its better stability in vivo as compared to PLLA and lower pro-inflammatory properties^32^.

We chose for the study pH 8 because sc-PLA is dedicated for oral administration. In vivo after mixing with food and secretions in the upper part of digestive tract scPLA enters the duodenum (small intestine) where the pH changes to 7.0–8.5. In the fasted state pH in the distal ileum range between 6.5 and 8^33–35^.

There has been no report concerning the in vivo biosafety of stereocomplexed PLA microparticles. The only data describe the in vivo activity of stereocomplexed hydrogels^36^, gels^37^, and nanofibers^38^. Therefore, the in-depth evaluation of in vivo biocompatibility of stereocomplexed microparticles in conjunction with the assessment of kinetic of extract release as well as its biological–immunomodulatory activity are highly required.

## Results and discussion

This work aimed to prepare biocompatible polymeric microparticles able to withstand the conditions in the colon. In this regard, stereocomplexed PLA microparticles are great candidates due to better hydrolytic stability than enantiomeric ones when the macromolecules are not functionalized with pH-responsive end groups. The first step was the synthesis of the desired PLLA and PDLA by ROP of lactide with the catalytic/initiating system composed of triflic acid and hexane-1,6-diol. Two sets of macromolecules were obtained: low molecular mass (lmm) with *M*_n_ of 3000 g/mol and medium molecular mass (mmm) with *M*_n_ of 7500 g/mol, as shown in Figs. S1–S3. These PLAs were subsequently used for the preparation of microparticles. The mixing of an equimolar amount of PLLA and PDLA induces stereocomplexation between polymeric chains, and as a result network composed of hydrogen bonds can be formed^7^. To achieve this aim, PLLA and PDLA were dissolved in THF (with or without studied *S. cadmica* extracts) to prepare stereocomplexed microparticles. Subsequently, Attenuated Total Reflectance-Fourier Transform Infrared (ATR-FTIR) analysis was employed to prove the formation of stereocomplex assemblies Fig. S4. The band at 909 cm^−1^ appeared after mixing PLLA and PDLA, which is responsible for the helical conformation of the stereocomplex^37^. Subsequently, DSC can be used to assess the thermal properties of the obtained materials Figs. S5, and S6. This technique can provide information about the purity of the obtained stereocomplexed microparticles since it shows the melting of even a small amount of homocrystallites. Among all tested samples only for sc-PLA-SCAPE (lmm), the presence of a small amount of homocrystallites was observed in the first heating run of the differential scanning calorimetry (DSC). In the second heating run, the stereocomplex crystallites were observed exclusively. Moreover, the effect of molar mass on the stereocomplexed microparticles melting temperature (*T*_m_) was observed, and those composed of medium molar mass exhibit* T*_m_ approximately 20 °C higher than stereocomplexes obtained by mixing of low molecular mass PLLA and PDLA. In addition, the decomposition temperature (*T*_d_) is also higher for stereocomplexed microparticles made of medium molar mass enantiomers. Interestingly, the *T*_d_ of obtained microparticles increased after the addition of *S. cadmica* extracts (Tables S1, S2). This result confirmed that the presence of extracts improved a heat resistance of the resulting microparticles.

**Figure 1:**
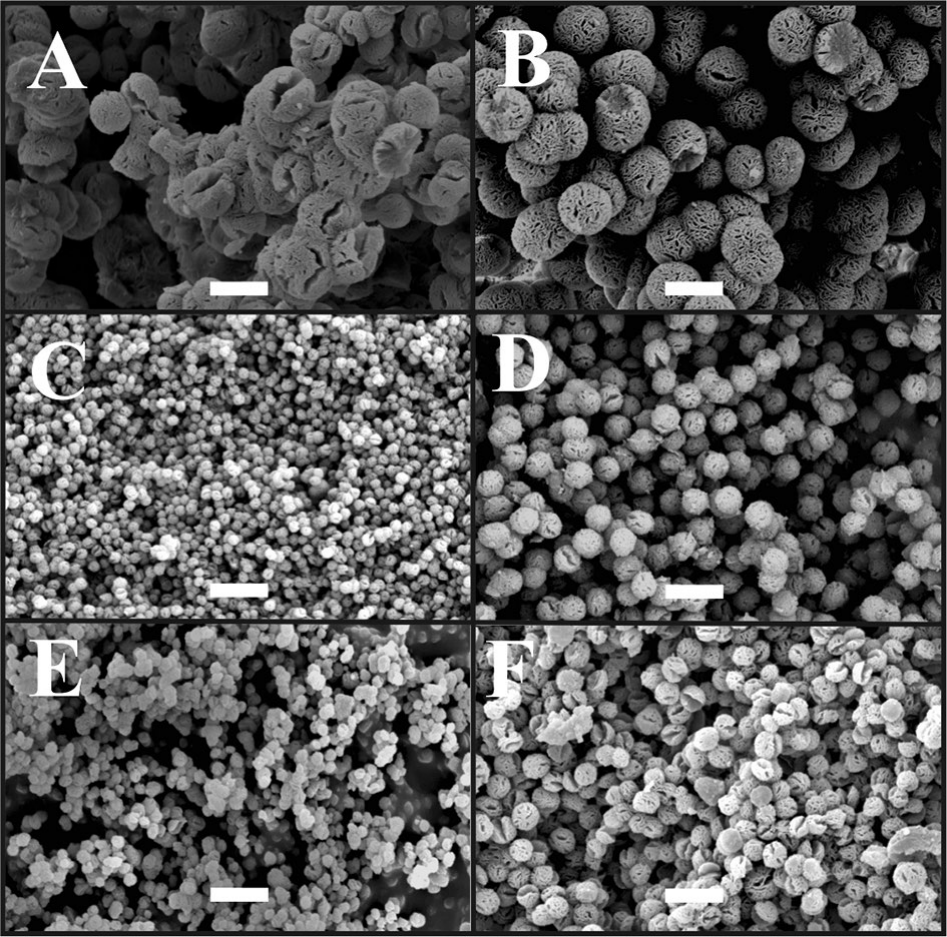
Exemplary scanning electron microscopy (SEM) microphotographs of the obtained stereocomplexed microparticles with low molecular mass—sc-PLA (lmm) (**A**) or with medium molecular mass—sc-PLA (mmm) (**B**), which were loaded with *S. cadmica* aerial part extract—sc-PLA-SCAPE (lmm) (**C**) and sc-PLA-SCAPE (mmm) (**D**) or with *S. cadmica* root extract sc-PLA-SCRE (lmm) (**E**) and sc-PLA-SCRE (mmm) (**F**). The scale bar denotes 5 μm.

Scanning electron microscopy (SEM) is an excellent tool for acquiring information about the morphology of the obtained microparticles. The spherical morphology is typical for stereocomplexed microparticles, as shown in Fig. 1. All microparticles were porous with a visible amount of cracks. Interestingly, the diameter of the blank microparticles was close to 5 µm, whereas there was a high decrease in their size to 1–2 µm after the addition of *S. cadmica* extracts. This observed decrease in size can be related to the interactions of PLLA and PDLA macromolecules with plant extracts or the presence of dimethyl sulfoxide (DMSO) during their self-assembly. For instance, the encapsulation of doxorubicin (DOX) in stereocomplexed microparticles causes a decrease in particle size from µm to nm, as shown in our previous work^9^. However, the additional factor that may influence the self-assembly process is the addition of DMSO, which enhances the solubility of the extracts. Even a small amount of solvent such as DMSO may change the dynamics of H-bond formation^39^ and the size of the resulting microparticles^40^. Therefore, these two substances are responsible for variation in the process of PLA macromolecule self-assembly since both can disrupt the formation of H-bonds.

### In vitro validation of cellular effects of stereocomplexed microparticles unloaded or loaded with *S. cadmica* extracts

Bio-polymers for medical applications have had to meet cytocompatibility and genotoxicity criteria in vitro, even in the early stages of composite optimization (ISO 10993-5:2009). Our microparticles are made of enantiomeric PLA that can form stereocomplexed microparticles. PLA is a biocompatible and biodegradable polymer approved by Food and Drug Administration^41,42^. Thus, PLA has been explored regarding many therapeutic applications, including antigen and drug delivery vehicles^41–43^.

**Figure 2:**
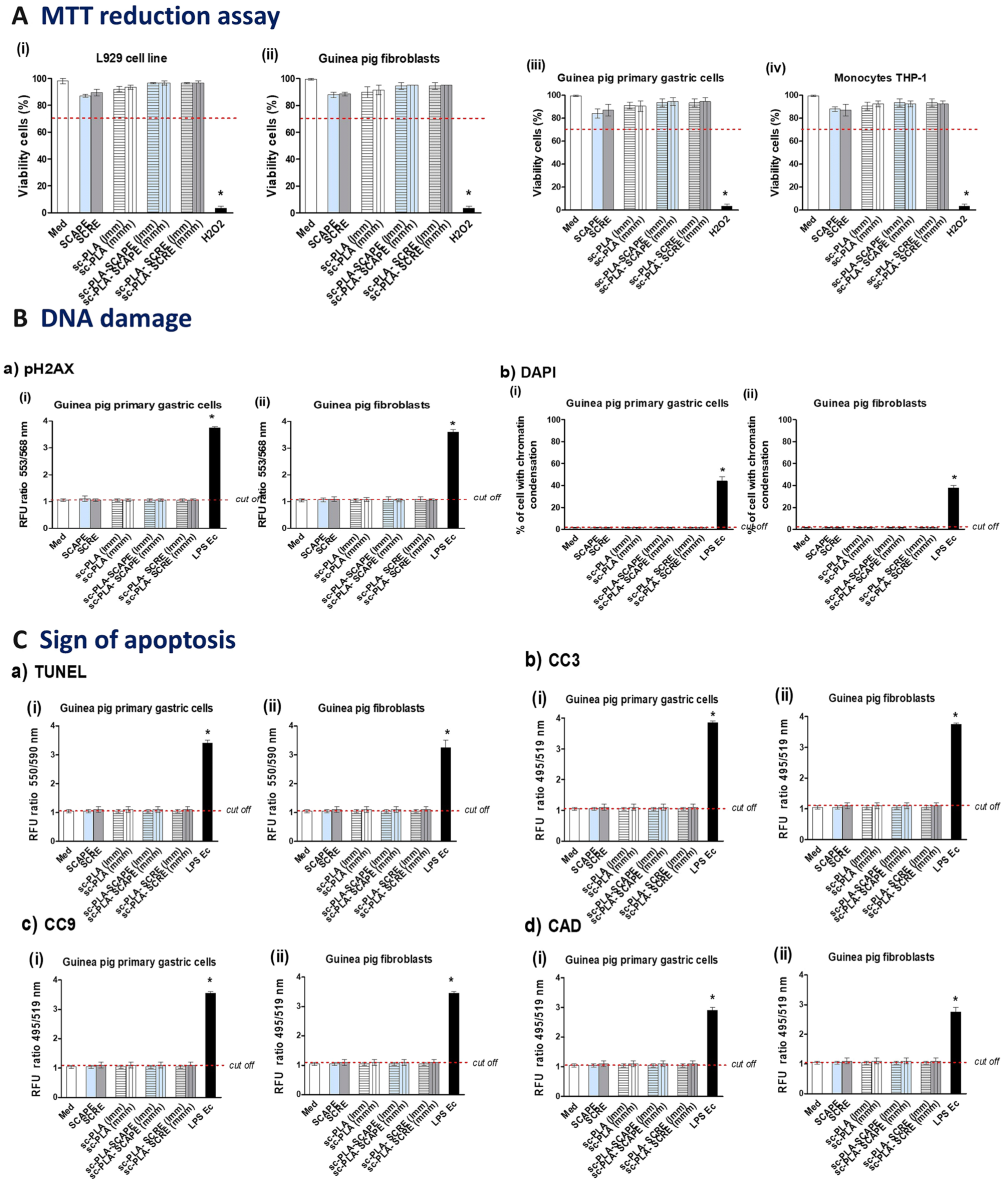
In vitro biocompatibility of *S. cadmica* extracts and stereocomplexed microparticles unloaded or loaded with these plant extracts. (**A**) Cell viability of (i) mouse fibroblasts L929, (ii) primary gastric epithelial cells of guinea pig, (iii) guinea pig fibroblasts, (iv) human THP-1 monocytes, evaluated by 3-(4,5-dimethylthiazol-2-yl)-2,5-, diphenyltetrazolium bromide (MTT) reduction assay. Complete RMPI-1640 medium (cRPMI) was used as a positive control (PC) of cell viability (100% viable cells) and 0.03% H_2_O_2_ as a negative control (NC) of cell viability (100% dead cells). The blue line indicates the minimal percentage of viable cells (70%) required to confirm the biomaterial as non-cytotoxic in vitro. (**B**) DNA damage: (a) detection of phosphorylated pH2AX by immunofluorescence, (b) chromatin condensation was evaluated by (4ʹ,6-diamidyno-2-fenyloindol)—DAPI staining of cell nuclei in gastric epithelial cells (i) and fibroblasts (ii). (**C**) Apoptosis of guinea pig primary gastric epithelial cells or guinea pig fibroblasts assessed by fluorescence assays: (a) terminal deoxynucleotidyl transferase dUTP nick end labeling—TUNEL, (b) caspase 3—CC3, (c) caspase 9—CC9 and (d) carbamoyl-phosphate synthetase 2 asparate transcarbamylase, and dihydroorotase—CAD in gastric epithelial cells (i) or fibroblasts (ii). Lipopolysaccharide (LPS) of *E. coli* (1 µg/mL) served as a positive control. Intensity of fluorescence was measured using a multifunctional SpectraMax i3 reader (Molecular Devices, San Jose, CA, USA) and presented as relative fluorescence units (RFU) ratio. Statistical significance: *p < 0.05; *untreated cells *vs.* cells treated with tested biomaterial. Data are presented as a median values ± range of four separate experiments (four independent experiments in triplicate for each experimental variant). Statistical significance: *p < 0.05; *untreated cells *vs.* cells treated with tested polymers. SCARE-*S. cadmica* root extract, SCAPE—*S. cadmica* aerial part extract; stereocomplexed microparticles: unloaded with *S. cadmica* extracts: sc-PLA (lmm); sc-PLA (mmm) or loaded with *S. cadmica* extracts: sc-PLA-SCAPE (lmm); sc-PLA-SCAPE (mmm); sc-PLA-SCRE (lmm); sc-PLA- SCRE (mmm).

In this study we assessed the biocompatibility of sterecomplexed microparticles empty or loaded with *S. cadmica* extracts. For in vitro assessment of the biocompatibility of tested microparticles we used the reference L929 mouse fibroblasts, which are recommended by ISO standards; furthermore, *Cavia porcellus*—guinea pig fibroblasts, guinea pig primary gastric epithelial cells and human THP-1 monocytes. The biocompatibility of tested formulations was evaluated on the basis of 3-(4,5-dimethylthiazol-2-yl)-2,5-,diphenyltetrazolium bromide (MTT) reduction assay, signs of DNA damage and apoptosis. The results on the safety of the biomaterials are presented in Fig. 2.

The *S. cadmica* extracts (SCAPE and SCARE) alone do not affect the cell viability (Fig. 2A). Similarly, stereocomplexed microparticles (sc-PLA), empty or loaded with *S. cadmica* extracts, were safe to L929 cells as well as guinea pig fibroblasts, guinea pig primary gastric epithelial cells and human THP-1 monocytes because the cell viability in MTT reduction assay was higher than 70% (Fig. 2A).

To exclusion the potential risk of genotoxicity of the studied formulations we used guinea pig primary gastric epithelial cells and fibroblasts treated with *S. cadmica* extracts alone, empty microparticles alone or microparticles loaded with *S. cadmica* extracts: sc-PLA (lmm), sc-PLA (mmm), sc-PLA-SCAPE (lmm); sc-PLA-SCAPE (mmm); sc-PLA-SCRE (lmm); sc-PLA-SCRE (mmm). We assessed the presence of phosphorylated molecule gamma H2A.X (phospho-Ser139), which is induced in response to DNA double strand breaks and supported this procedure by staining cell nuclei with 4ʹ,6-diamidyno-2-fenyloindol (DAPI). We showed no increase in DNA damage in cells treated with *S. cadmica* extracts alone, microparticles alone or microparticles loaded with *S. cadmica* extracts as compared to control cells (Fig. 2B).

Our results are in line with the study developed by Uzun et al*.,* excluding PLA genotoxicity using Chinese Hamster Ovary (CHO-K1) cell line by comet assay and cytokinesis-blocked micronucleus (CBMN) assay^44^.

Programmed cell death—apoptosis occurs during the development and ageing of cells, and provide maintaining global homeostasis in the host organism^44–46^. Upregulation of apoptosis can affect the equilibrium between cell growth and cell death, resulting in organ dysfunction^47^. We checked whether plant extracts and sc-PLA (lmm); sc-PLA (mmm); sc-PLA-SCAPE (lmm); sc-PLA-SCAPE (mmm); sc-PLA-SCRE (lmm) or sc-PLA-SCRE (mmm) do not increase cell apoptosis. In four different assays, including: terminal deoxynucleotidyl transferase dUTP nick end labeling—TUNEL assay, estimation of caspase 3—CC3, caspase 9—CC9 and carbamoyl-phosphate synthetase 2 asparate transcarbamylase, and dihydroorotase—CAD none of tested *S. cadmica* extracts and sterecomplexed microparticles, unloaded or loaded with plant extracts showed pro-apoptotic activity (Fig. 2C).

**Figure 3:**
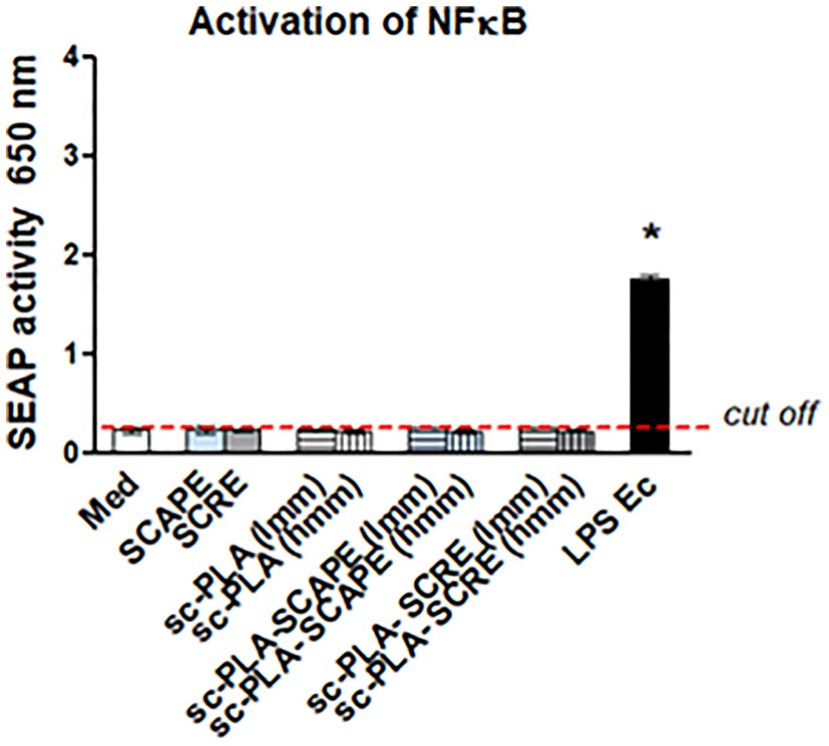
Activation of nuclear factor kappa B (NF-κB) in THP1-XBlue monocytes. Med-medium; SCAPE—*S. cadmica* aerial part extract; SCRE—*S. cadmica* root extract; sc-PLA (lmm) and sc-PLA (hmm)—sterocomplexed microparticles with low molecular mass or high molecular mass, respectively, unloaded or loaded with *S. cadmica* extracts; LPS Ec—lipopolysaccharide *Escherichia coli*. SEAP—secretory embryonic alkaline phosphatase. *The difference statistically significant; *p* < 0.05; LPS Ec *vs*. Med.

According to literature, PLA may induce inflammatory responses, due to its hydrophobicity^48^, and release of acidic degradation by-products^49,50^. In this study we evaluated the ability of *S. cadmica* extracts and stereocomplexed microparticles unloaded: sc-PLA (lmm), sc-PLA (mmm), sc-PLA-SCAPE (lmm) or loaded with *S. cadmica* extracts: sc-PLA-SCAPE (mmm); sc-PLA-SCRE (lmm); sc-PLA-SCRE (mmm) to activate in THP1-XBlue monocytes of nuclear factor kappa-B (NF-kappa B) signaling pathway, on the basis of the release of secretory embryonic alkaline phosphatase—SEAP (Fig. 3). The concentration of SEAP was similar in cell cultures with tested samples *vs.* control cells sub-cultured in culture medium alone (negative control), while cells treated with lipopolysaccharide (LPS) *Escherichia coli* (positive control), which is recognized by toll-like receptor (TLR)-4 on NF-kappa B pathway, responded by the significant SEAP production. Strong activation of monocytes by the components of different biomaterials alone, biologically active substances used for encapsulation or contaminants such as endotoxin may induce acute inflammation, potentially resulting in pus formation, tissue degradation, and disintegration of cell barrier^51^. According to the Food and Drug Agency Guidance as well as the European Medicines Agency, an endotoxin content cannot be higher than 0.25 EU in biomaterials being in contact with human blood/tissue. Only controlled inflammation leads to revascularization and regeneration of injured tissue. *S. cadmica* extracts or microparticles used in this study did not show pro-inflammatory activity. This observed behavior could be attributed to the enhanced stability of stereocomplexed microparticles in vivo in comparison to enantiomeric components. It was shown^52^ that degree of inflammatory reaction is correlated with the bulk size of sc-PLA nanofibers mats after implantation. The stereocomplexed nanofibers’ morphology, crystallinity, and molar mass were unchanged after 12 weeks, whereas significant breakdown was observed for enantiomeric PLAs. Therefore, it can be anticipated that cross-linking of PLLA and PDLA chains suppresses the hydrolysis of molecular chains in vivo, and the inflammatory reaction is limited^52^.

Activation of NF-kappa B was evaluated in cells incubated for 24 h with *S. cadmica* extract alone (root-SCRE or aerial part-SCAPE), stereocomplexed microparticles with low molecular mass—sc-PLA (lmm) or with medium molecular mass—sc-PLA (mmm), or such microparticles loaded with *S. cadmica* aerial part extract—sc-PLA-SCAPE (lmm); sc-PLA-SCAPE (mmm) or root extract—sc-PLA-SCRE (lmm); sc-PLA-SCRE (mmm) on the basis of secreted embryonic alkaline phosphatase (SEAP). Cells in culture medium alone (Med) were used as a negative control, while monocytes stimulated with lipopolysaccharide (LPS) of *E. coli* (Ec) served as a positive control. Data are presented as median values ± range of four separate experiments (four independent experiments in triplicate for each experimental variant). Statistical significance: *p < 0.05; *untreated cells and cells treated with tested polymers *vs*. cells treated with LPS Ec.

### Kinetics of *S. cadmica* extracts release from microparticles in different pH in vitro

**Figure 4:**
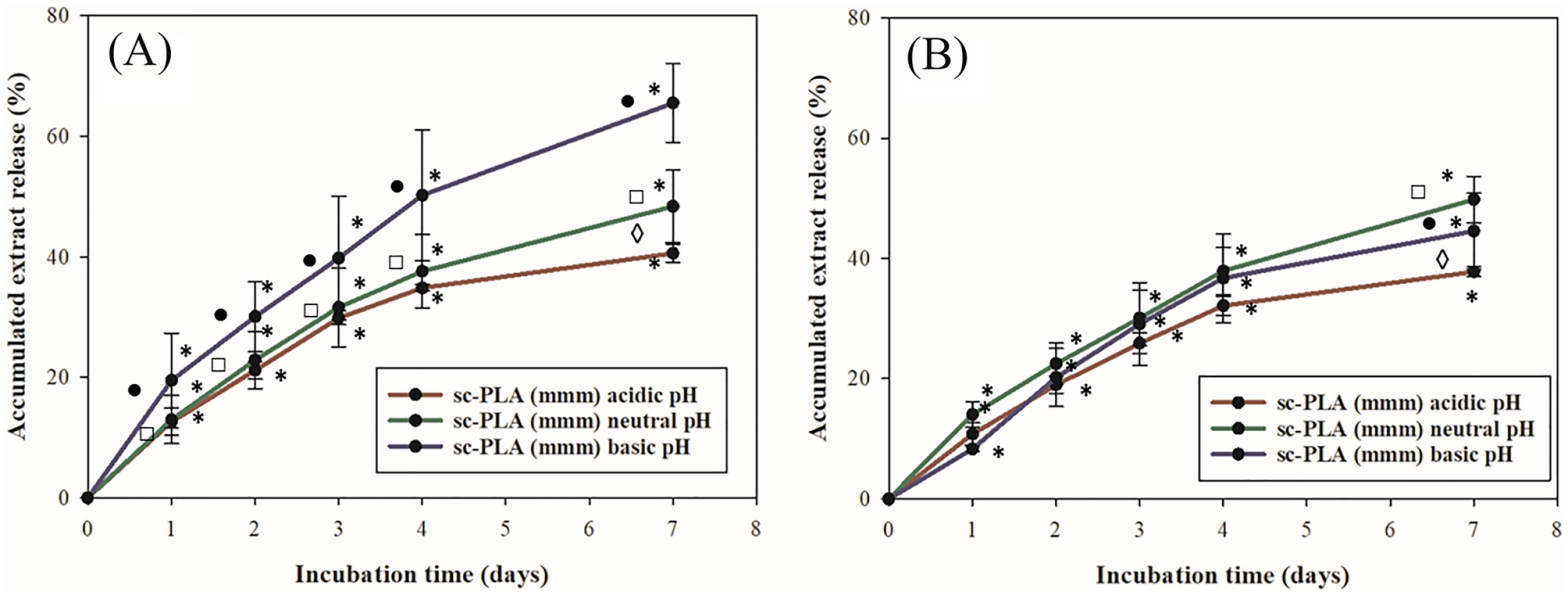
In vitro release profiles of extracts from stereocomplexed microspheres (sc-PLA), formed by multiple H-bonds between poly(l-lactide) (PLLA) and poly(d-lactide) (PDLA) chains. The sc-PLA with medium molecular mass (mmm) loaded with *S. cadmica* aerial part extract—SCAPE **(A)** or with *S. cadmica* root extract—SCRE **(B)**. Statistical significance for *p* < 0.05 in the non-parametric Kruskal–Wallis test, for extract release *according to the time: 1 *vs*. 2; 2 *vs*. 3; 3 *vs*. 4; 4 *vs*. 5; 5 *vs*. 6; 6 *vs*. 7 (days for each pH value separately); filled circle—basic pH *vs*. neutral pH according to the same time point; open square—basic pH *vs*. acidic pH according to the same time point; open diamond—neural pH *vs*. acidic pH according to the same time point.

Due to the fact that sc-PLA-SCAPE (mmm) or sc-PLA-SCRE (mmm) loaded with *S. cadmica* extracts (aerial part-SCAPE or root-SCRE) were investigated in vivo, the same set of microparticles was used to elucidate the extracts release rate in acidic (pH 5.5), neutral (pH 7.2), and basic (pH 8.7) conditions. The experiments were conducted for 7 days. It was observed that the release rate ranges from 37 to 70%, as shown in Fig. 4. In general, the slowest release is observed in acidic pH, whereas in neutral and basic pH, it depends on the type of encapsulated extract. The observed increase in release rate for sc-PLA-SCAPE (mmm) microparticles (Fig. 4A) than sc-PLA-SCRE (Fig. 4B) may be attributed to the localization of a high amount of extract on the surface of the particles. This is correlated with the higher initial burst release from these microparticles, which is in agreement with the literature examples^32,53^. Moreover, the porosity of the obtained microparticles may play a role in the difference in extracts’ release^54,55^.

It is worth noting that the most important time for extract release from microspheres is for 24 h or 48 h of their presence in the gut or small intestine. It can be observed that microspheres release 12.6% (acidic pH), 12.9% (neutral pH), and 19.5% (basic pH) of SCAPE after 24 h, whereas the amount of released SCRE was 10.8% (acidic pH), 14% (neutral pH), and 8.3% (basic pH). After 48 h of extracts release, the 21.1% (acidic pH), 22.6% (neutral pH), 30% (basic pH) for SCAPE and 18.9% (acidic pH), 22.5% (neutral pH), 20.2% (basic pH) for SCRE. It can be seen that there is only a slightly faster release of SCAPE; however, the difference is not significant. It can be anticipated that obtained microspheres would provide a prolonged release of extracts in the stomach^56^, due to the observed extracts release in vitro (Fig. 4). In addition, the size of obtained microparticles predisposes their uptake by a small intestine as it was shown by Reineke et al. for polystyrene particles^57^ therefore, those particles may reach the desired target.

It was particularly interesting to see if extracts would be released at a pH around 8, which is found in the intestine, where interaction of plant compounds with the mucosal lymphatic system can be expected. On the other hand, the release of extracts at acidic pH in the stomach may facilitate the direct action of the plant formulation on bacteria in this milieu^27^. Both effects can be useful in controlling *H. pylori* infection. To confirm the assumed effects of the studied extracts, further in vivo studies will be developed on the guinea pig model.

### In vivo biosafety of stereocomplexed microparticles unloaded or loaded with *S. cadmica* extracts

Particles made of PLA for potential medical applications should be characterized towards their interactions with the human tissues^58,59^. In this study, initial biosafety tests were performed on guinea pigs using stereocomplexed microparticles alone: sc-PLA (mmm) or such particles loaded with *S. cadmica* extracts: sc-PLA-SCAPE (mmm); sc-PLA-SCRE(mmm). For in vivo biosafety study the sc-PLA (mmm) were selected since the encapsulation of *S. cadmica* extracts using these microparticles was better than using sc-PLA (lmm) microparticles.

**Figure 5:**
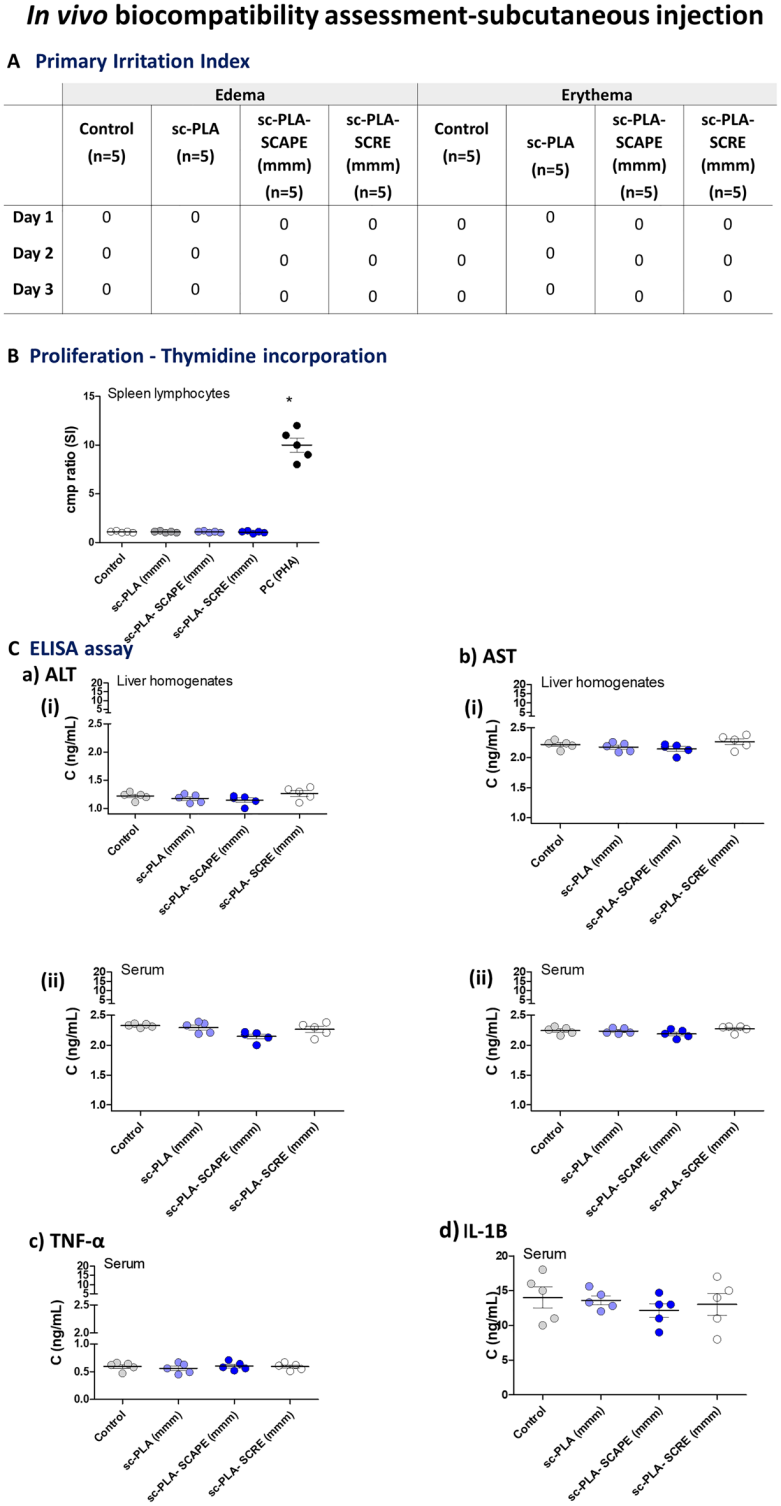
In vivo biocompatibility assessment of studied microparticles in guinea pigs injected subcutaneously. Tested *S. cadmica* extracts alone, stereocomplexed microparticles sc-PLA (mmm) alone or sc-PLA (mmm) loaded with *S. cadmica* aerial part extract—sc-PLA-SCAPE (mmm), *S. cadmica* root extract—sc-PLA-SCRE (mmm) or physiological saline were subcutaneously injected to guinea pigs. Guinea pigs were monitored for 72 h for adverse skin reactions **(A).** The appearance of edema and erythema was assessed and graded daily according to the Primary Irritation Index (PII) = 0/3, where 0 indicates that the irritation is negligible. At 72 h post-injection, the animals were terminated and spleen, liver and blood samples were collected for further evaluation. **(B)** Splenic lymphocytes were tested for proliferation towards mitogen—phytohemagglutinin (PHA) as positive control (PC) by (^3^H)-thymidine incorporation into cellular DNA. Stimulation index (SI) was calculated by dividing the radioactivity counts (cpm/min) of cells stimulated with *S. cadmica* extracts or microparticles by the radioactivity of cells in culture medium alone. (**C**) The level of alanine aminotransferase—ALT (**a**) and aspartate aminotransferase—AST (**b**) in liver homogenates (**i**) or in serum samples (**ii**); the level of tumor necrosis factor alpha—TNF-α (**c**) and interleukin (IL)-1B (**d**) in serum samples, detected by the enzyme linked immunosorbent assay (ELISA). Five animals per group were examined. The results are presented as median values ± range.

Subcutaneous injection of *S. cadmica* extracts, sc-PLA alone or sc-PLA loaded with *S. cadmica* extracts did not induce signs of skin irritation and edema or erythema in the vicinity of the site of injection during the course of the experiment, up to 72 h after subcutaneous injection (Fig. 5A). After subcutaneous injection of studied formulations selected tissues and organs: spleen and liver did not show signs of inflammation and splenocytes did not show an increased proliferative activity (Fig. 5B). The levels of aminotransferase (AST) or alanine aminotransferase (ALT) in the liver homogenates (Fig. 5Ca,b) and in serum samples (Fig. 5Cc,d) were similar in control animals and those injected with empty sc-PLA microparticles or such microparticles loaded with *S. cadmica* extracts. Similarly, there was no difference in the serum concentration of TNF-α and IL-1 B in control and tested animals.

**Figure 6:**
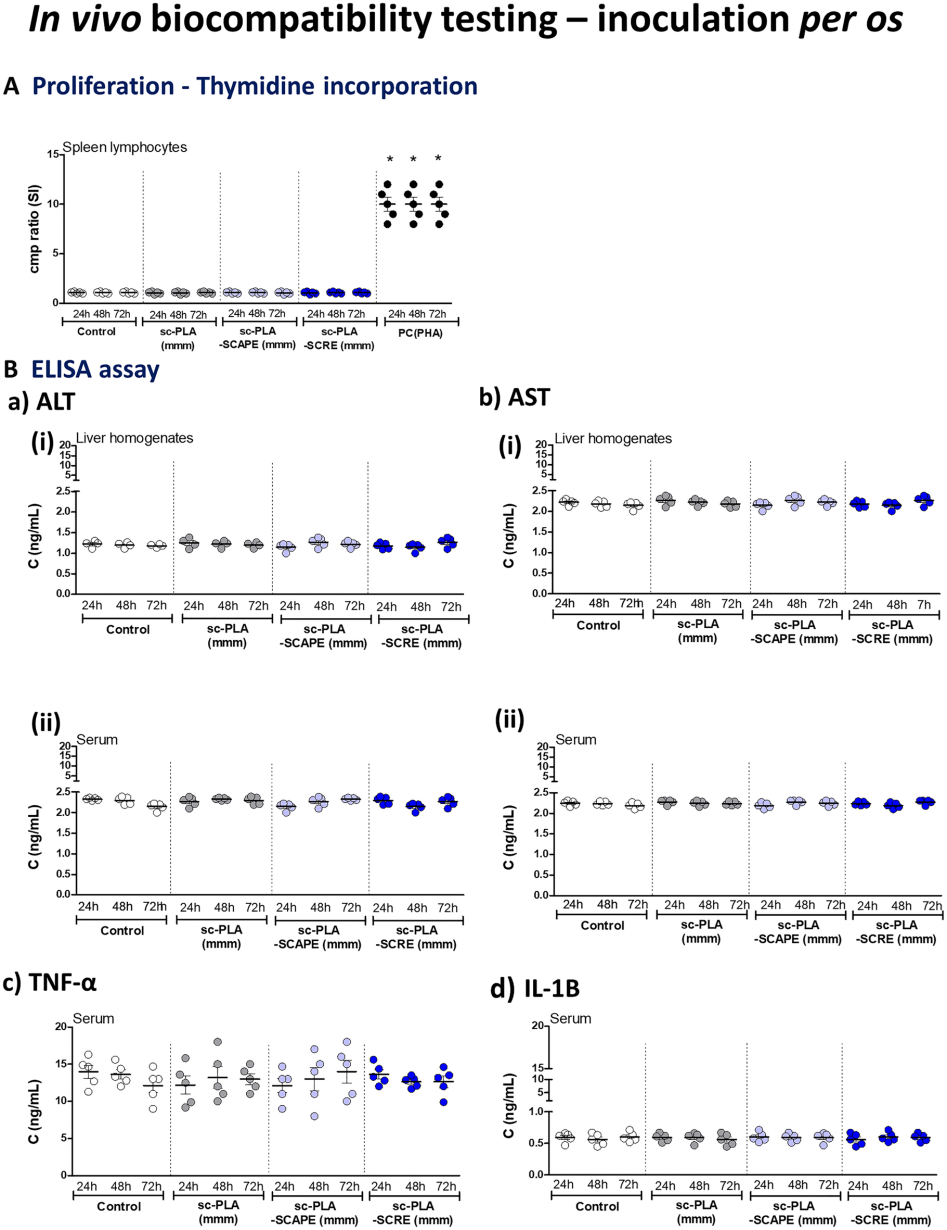
In vivo biosafety of tested stereocomplexed microparticles sc-PLA with medium molecular mass—sc-PLA (mmm) alone or such particles loaded with *S. cadmica* aerial part extract—sc-PLA-SCAPE (mmm) or *S. cadmica* root extract—sc-PLA-SCRE (mmm) after oral administration to guinea pigs. At 24, 48 and 72 h after inoculation of animals with tested formulations or physiological saline (control animals) the animals were terminated and spleen, liver and blood samples were collected for further evaluation. **(A)** Splenic lymphocytes were tested for proliferation towards mitogen—phytohemagglutinin (PHA) as positive control (PC) by (^3^H)-thymidine incorporation into cellular DNA. Stimulation index (SI) was calculated by dividing the radioactivity counts (cpm/min) of cells stimulated with *S. cadmica* extracts or microparticles by radioactivity of cells in culture medium alone. (**B**) The level of alanine aminotransferase—ALT (**a**) and aspartate aminotransferase—AST (**b**) in liver homogenates (i) or serum samples (ii); the level of tumor necrosis factor alfa (TNF-α) (**c**) and interleukin (IL)-1B (**d**) in serum samples, detected by the enzyme linked immunosorbent assay- ELISA assay. Five animals per group were examined. The results are presented as median values ± range.

Furthermore, testing of proliferation of splenocytes, the levels of ALT and AST in the liver homogenates and serum samples as well as serum level of TNF-α/IL-1 B were performed after *per os* inoculation of guinea pigs with sc-PLA (mmm), empty or loaded with *S. cadmica* extracts (Fig. 6). There was no difference between studied biomarkers in control animals vs. animals inoculated with the microparticles.

### Influence of *S. cadmica* extracts on activity of guinea pig bonne marrow derived macrophages

Considering *per os* application of stereocomplexed microparticles loaded with *S. cadmica* extracts for increasing an immune response towards gastric pathogen *H. pylori* in this study we asked whether these extracts are able to increase an antibacterial activity of guinea pig bone marrow derived macrophages (BMDM). Macrophages are essential players in the maintenance of intestinal homeostasis and in stimulation of immunity in the gut^60^. The major activities of macrophages include the ability to engulf and digest infectious agents, present their antigenic determinants to T lymphocytes and to deliver proinflammatory cytokines^61^. The bone marrow derived macrophages are a good experimental model since they can differentiate into mature macrophages in vitro^61^. It has been shown that polyphenols, biologically active components, increased the number of T helper 1 (Th1) lymphocytes, natural killer (NK) cells, macrophages and dendritic cells (DCs) in Peyer’s patches and spleen in C3H/HeN mice, after oral administration of polyphenols extracted from fruits^28^. In humans, the number of regulatory T lymphocytes can be boosted by polyphenols^29,30^. During *H. pylori* infection an inflammatory response in the gastric mucosa is elevated and becomes chronic, and due to this the potential therapeutic compound should be selected taking into account its pro- or anti-inflammatory activity. In this study we used *S. cadmica* aerial part and root extracts enriched in polyphenols to pulse BMDM for 24 h (induction), to see whether priming of cells with these plant extracts will result in upregulation of phagocytic activity of macrophages in conjunction with an increased expression of CD11b integrin, involved in phagocytosis, as well as secretion of proinflammatory TNF-α or anti-inflammatory IL-10. We also checked whether restimulation of cells with the same extract for 5 days will result in stronger macrophage response. Furthermore, we wanted to know how cells primed with *S. cadmica* extracts will react to restimulation with *H. pylori*. Finally, whether additional restimulation of cells for 24 h with *S. cadmica* extract will potentially neutralize an inhibitory effect driven by *H. pylori*. Previously we showed that *H. pylori* using their surface haemagglutinins and LPS inhibit phagocytosis^19,21^. Also Allen et al., showed that these bacteria may survive intracellularly in megasomes^20^. In this study we assessed the ability of guinea pig BMDM to engulf fluorescently labeled *E. coli* to see whether the ingestion can be upregulated in response to *S. cadmica* extracts.

**Figure 7:**
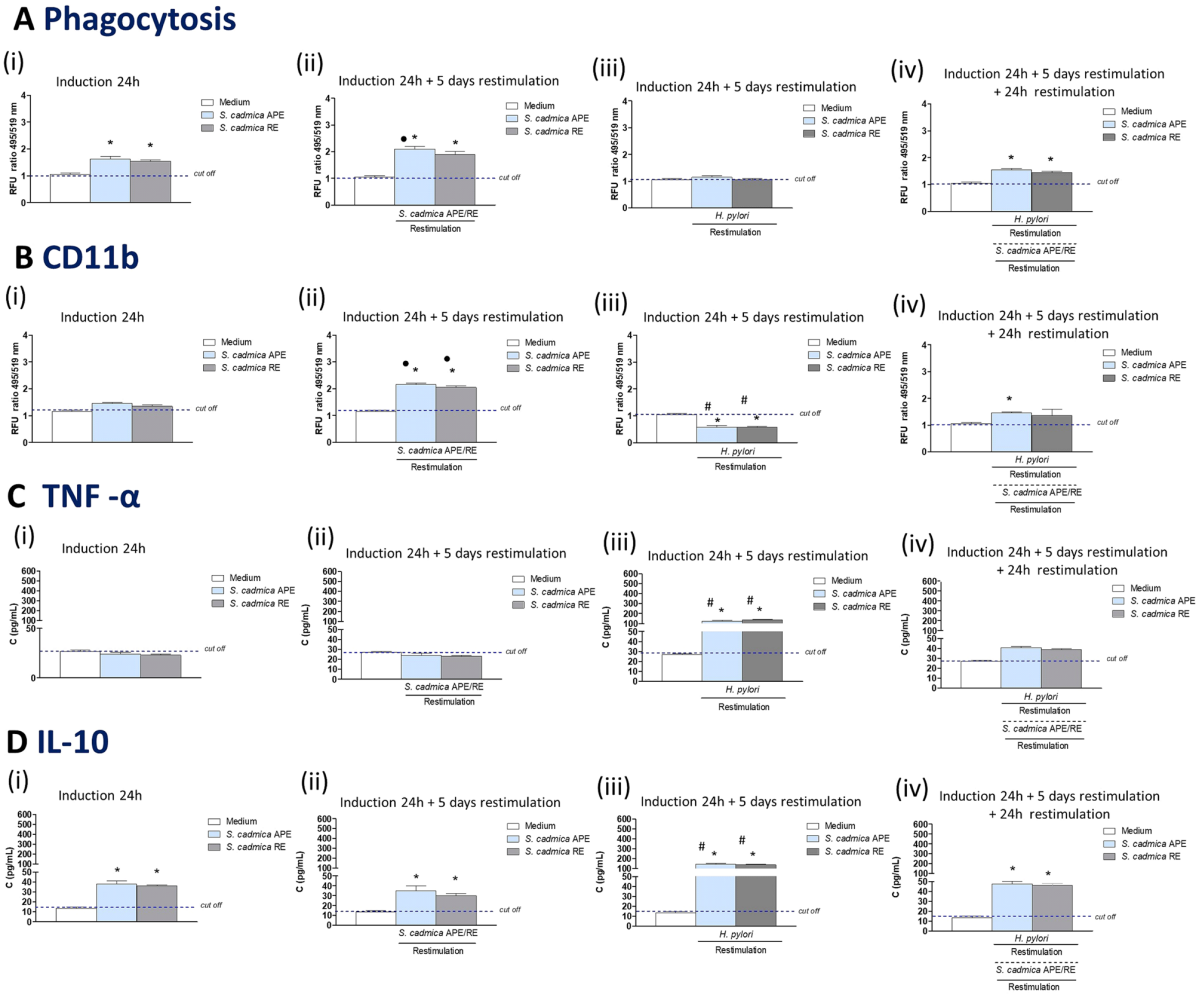
Increased phagocytic activity and downregulation of secretory activity of guinea pig bone marrow macrophages primed and then restimulated with *S. cadmica* extracts in vitro. The guinea pig bone marrow derived macrophages (BMDM) were treated with *S. cadmica* aerial part (APE) or roots (RE) extracts alone for 24 h (i), restimulated for 5 days with the homologous plant extract (ii) or *H. pylori* (iii), and for additional 24 h with *S. cadmica* extract (iv). (**A**) Phagocytosis towards *Escherichia coli* was determined on the basis of relative fluorescence units (RFU) ratio in relation to phagocytic activity of control cells in culture medium alone. The results are presented as median relative fluorescence units (RFU) ratio ± range. (**B**) Deposition of CD11b cell surface integrin was determined by the immunofluorescence staining of cells with anti-CD11b antibodies and showed as median RFU ratio ± range. The fluorescence intensity was measured in a MultiscanEX reader at 495 nm (excitation) and 519 nm (emission). (**C**) The level of tumor necrosis factor (TNF)-α and (**D**) interleukin (IL-10), was assessed in cell culture supernatants by the enzyme linked immunosorbent assay—ELISA (pg/mL), and presented as median values ± range. Statistical significance for p < 0.05 in the non-parametric Mann–Whitney *U* test. *Cells treated stimulators vs. control cells in culture medium alone; filled circle—cells induced with *S. cadmica* extract (24 h) vs. cells induced *with S. cadmica* extract (24 h) and restimulated with *S. cadmica* extract (5 days); #cells induced with *S. cadmica* extract (24 h) vs. cells induced with *S. cadmica* extract (24 h), restimulated for 5 days with *H. pylori*; #cells induced with *S. cadmica* extract (24 h) vs. cells induced with *S. cadmica* extract (24 h), restimulated for 5 days with* H. pylori.*

Phagocytic activity of BMDM is demonstrated in Fig. 7A. Macrophages primed with *S. cadmica* aerial part or root extract (24 h), and then restimulated with the homologous extract for 5 days, showed an increased phagocytic activity towards *E. coli* (Fig. 7Ai,ii). The phagocytic activity of macrophages primed with *S. cadmica* extracts followed by restimulation of cells for 5 days with *H. pylori* was diminished (Fig. 7Ai,ii,iii). However, an additional restimulation of macrophages with *S. cadmica* extract for 24 h neutralized the negative effect driven by *H. pylori* (Fig. 7Aiv). Also other researchers have shown that plant extracts can enhance the phagocytosis process, which is consistent with results obtained in this study^62–64^.

Beneficial effect of *S. cadmica* extracts in the course of phagocytosis might be related to enhanced expression of macrophage CD11b adhesin molecules (Fig. 7B), which promote the interaction of macrophages with endothelial cells and function as complement receptors increasing complement dependent engulfment of infectious agents^65–67^. In the current study an increased expression of CD11b on BMDM was demonstrated 6 days after stimulation of cells with *S. cadmica* extracts (Fig. 7Bi,ii), which means that potentially also another factors may drive the enhancement of macrophage phagocytic activity in response to *S. cadmica* extracts. It is interesting that restimulation with *H. pylori* of cells, which were primed with *S. cadmica* extracts, resulted in diminished deposition of CD11b, which was compatible with diminished phagocytic activity of macrophages (Fig. 7Biii). However, additional restimulation of macrophages with *S. cadmica* extracts upregulated the expression of CD11b, which was related to elevated macrophage phagocytic activity (Fig. 7Biv).

Infection with *H. pylori* is correlated with the chronic inflammatory response in the gastric tissue. Inflammation is under control of pro-inflammatory and anti-inflammatory cytokines delivered by gastric epithelial cells, endothelium and immunocompetent cells, including macrophages. In this study we assessed whether *S. cadmica* extracts are able to neutralize proinflammatory cytokine—TNF-α, induced by *H. pylori*. We showed that *S. cadmica* extracts alone did not increase the production of TNF-α after priming of cells or priming of them and then restimulation (Fig. 7Ci,ii). However, *S. cadmica* extracts effectively diminished the production of this pro-inflammatory cytokine, which was upregulated in macrophages restimulated with *H. pylori* (Fig. 7iii,iv).

*S. cadmica* extracts induced the production of IL-10 by macrophages, which were exposed to plant formulation for 24 h and restimulated for 5 days with the homologous extract (Fig. 7Di,ii). Restimulation of macrophages, which were primed for 24 h with *S. cadmica* extracts, with *H. pylori* for 5 days resulted in further enhancement of IL-10 production (Fig. 7Diii). In the previous study we showed that LPS *H. pylori* due to induction of intense IL-10 production by immune cells diminished the expansion and cytotoxic activity of natural killer cells, which together with granulocytes and macrophages consist the first line of natural immune defense^22^. In the current study the enhanced production of IL-10 by macrophages in response to *H. pylori* was diminished after an additional restimulation of cells for 24 h with *S. cadmica* extracts (Fig. 7iv).

The immunomodulatory properties of *S. cadmica* extracts resulting in an enhancement of phagocytic activity of macrophages and diminishing of *H. pylori* driven TNF-α or IL-10 production potentially may help to control *H. pylori* infection as well as related chronic inflammatory response. Flavonoids present in the studied *S. cadmica* extracts might be responsible for diminishing *H. pylori* driven production of investigated cytokines by macrophages and elevated phagocytic activity. Similar effects of flavonoids present in different plant extracts have been shown by other researchers on various cell culture models in vitro^68–72^. Thus stereocomplexed microparticles (sc-PLA) loaded with *S. cadmica* extracts potentially may deliver their immunomodulatory components to the gut, where they can increase phagocytic activity of macrophages in conjunction with enhancement of CD11b expression as well as regulation of TNF-α and IL-10 secretion.

Further in vivo study is needed on the guinea pig model, which was well characterized by us in terms of an immune response during *H. pylori* infection^73^, to see whether *S. cadmica* extracts delivered by sc-PLA will help to eliminate or prevent ongoing experimental infection with these bacteria, and control an inflammatory response.

## Conclusions

In this study we developed method of encapsulation of *S. cadmica* extracts, enriched with polyphenols with immunomodulatory activity, in stereocomplexed microparticles (sc-PLA). We showed that obtained polymers are safe in vitro and in vivo. *S. cadmica* extracts enhanced the phagocytic activity of guinea pig bone marrow-derived macrophages, which was diminished in response to *H. pylori*, and neutralized *H. pylori-*driven enhanced production of TNF-α and IL-10. Therefore, *S. cadmica* extracts encapsulated in sc-PLA can be recommended for further in vivo study to confirm their role in supporting the immune response towards *H. pylori* in guinea pigs experimentally infected with these bacteria.

## Materials and methods

### Chemicals

l,l-lactide and d,d-lactide (99%, Purac, Gorkum, Netherlands) were consecutively crystallized from dry 2-propanol and purified just before use by sublimation in vacuo (10^−3^ mbar, 85 °C). Triflic acid (trifluorometanosulfonic acid, 98% Merck, Darmstadt, Germany) was used without further purification. Methylene chloride was distilled over P_2_O_5_ and stored under a vacuum over molecular sieves. Methanol, 2-propanol and THF (POCH, Gliwice, Poland, pure p.a. grade) and hexan-1-6-diol (Sigma Aldrich, Saint Louis, Missouri, USA), sodium acetate (99%, Merck, Darmstadt, Germany), acetic acid and CaO (98%, Merck, Darmstadt, Germany) were used as received. Phosphate buffer saline (pH 7.4, 0.1 M) and Tris buffer (pH 8.7, 0.1 M) were purchased from Merck (Darmstadt, Germany) in powder form and prepared according to the manufacturer’s instructions. Acetate buffer (pH 5.5, 0.1 M) was prepared as follows: 7.721 g of sodium acetate, and 352 mg of glacial acetic acid was dissolved in 1L of water.

### Characteristics of* Salvia cadmica* extracts

*S. cadmica* plants were grown from seeds collected in 2017 in the Garden of Medicinal Plants, Wroclaw Medical University (Wroclaw, Poland). The seeds were sewn in March 2018 in the soil. The obtained plants were cultured in the Garden of Medicinal Plants, Medical University of Lodz (Lodz, Poland) (51° 77ʹ N, 19° 49ʹ E) in the Garden of Medicinal Plants, Medical University of Łodz (Lodz, Poland) (51° 77ʹ N, 19° 49′ E) and authenticated by E. Piątczak. A voucher specimen (no EP-SC1-2018) was deposited at the Department of Biology and Pharmaceutical Botany, Medical University of Lodz (Lodz, Poland) were used in this study. *S. cadmica* name has been checked with “World Flora Online” (www.worldfloraonline.org), WFO (2022): *Salvia cadmica* Boiss. Published on the Internet; http://www.worldfloraon line.org/taxon/wfo-0000300534. Accessed on: 31 Dec 2022.

Hydromethanolic (80% v:v) *Salvia cadmica* Boiss. (extracts derived from lyophilized and powdered roots and aerial parts of 6-month-old plants) was preparation and prepared their chemical characteristic by using high-performance liquid chromatography (HPLC) as previously described^74^. Detailed method of extracts preparation and their chemical characteristic have been described previously^74^.

All experiments on plants (either cultivated or wild), including the collection of plant material, were performed in accordance with the institutional, national, and international guidelines and legislation.

### Polylactide synthesis

The PLLA and PDLA enantiomeric polymers were synthesized by ring-opening polymerization (ROP) of appropriate lactide^75^. L,L-LA (4.6 g, 31.9 mmol) was placed in a Schlenk tube and degassed under reduced pressure. After filling the reactive vessel with argon, methylene chloride (15 mL) was added via a syringe. After the complete dissolution of the lactide, hexane-1,6-diol (1.59 or 3.18 mmol) and trifluoromethanesulfonic acid (0.43 mmol) were introduced through the rubber septum. Polymerization was conducted at RT for 24 h; then a sample was withdrawn for SEC analysis. The remaining polymerization mixture was diluted with methylene chloride and CaO was added to neutralize trifluoromethanesulfonic acid. CaO was filtered off, the solution was precipitated in cold methanol, and the obtained product was dried under vacuum. The purified homopolymer was characterized by ^1^H NMR.

^1^H NMR (CDCl3): δ = 5.16 (q, 1H, CH–CH_3_ polymer), 4.36 (q, 1H, CH–CH_3_ end-group polymer), 4.13 (m, 2H, –C(O)–O–CH_2_–CH_2_–CH_2_–CH_2_–CH_2_–CH_2_–C(O)–hexane-1,6-diol end group), 1.58 (d, 3H, CH_3_ polymer), 1.66 (m, 4H, –C(O)–O–CH_2_–CH_2_–CH_2_–CH_2_–CH_2_–CH_2_–C(O)–hexane-1,6-diol), 1.27 (m, 4H, –C(O)–O–CH_2_–CH_2_–CH_2_–CH_2_–CH_2_–CH_2_–C(O)–hexane-1,6-diol) ppm.

### Synthesis and characterization of microparticles and encapsulation of extracts

The spontaneous precipitation was used to prepare stereocomplexed microparticles (MPs)^10^. To achieve this aim, 100 mg of PLLA and 100 mg of PDLA were dissolved separately in 4 mL of tetrahydrofuran (THF) and mixed without stirring. The interactions between enantiomeric chains lead to the precipitation of polymers in the form of microparticles. Finally, the supernatant was removed, the microparticles were washed with THF and dried under a vacuum. The sterocomplexed microparticles were loaded with the SCAPE or SCRE extract as follows: 100 mg of PLLA and 100 mg of PDLA were separately dissolved in 4 mL of THF. To dissolve 10 mg extracts the 10 mL water/ethanol (7:3) was required and 400 µL of the extract solution was added to the solution of one enantiomer along with 0.5 mL of dimethyl sulfoxide (DMSO) to enhance the miscibility with polymeric solution. After 2 h, the solutions were mixed and left at room temperature (25 °C) without stirring. The precipitation of microparticles was observed after 24 h. Subsequently, the supernatant was removed by decantation, the solid was washed with three portions of fresh THF and dried at room temperature under vacuum. The prepared MPs contained 10 µg extracts/1 mg MPs or 42 µg extracts/1 mg MPs, respectively. The prepared blank and microparticles loaded with *S. cadmica* extracts were analyzed by scanning electron microscope (SEM), differential scanning calorimetry (DSC), thermogravimetric analysis (TGA), and Fourier transform infrared spectroscopy (FTIR), as described previously^10^.

### Encapsulation efficiency of extracts

The amount of encapsulated extracts was determined by the UV/Vis method. 10 mg of the sc-PLA-loaded with *S. cadmica extract* was dissolved in 10 mL of DMSO/CHCl_3_ solution (2:5). DMSO was used to destroy the H-bond between the helix of PLA enantiomers to complete dissolution of sc-PLA microparticles and release all encapsulated and physically absorbed on the surface extract to the solution. After that, the UV/Vis measurement was performed at the wavelength of 324 nm. The amount of extracts was calculated from the calibration curve (Fig. S8). The measurement can be performed without separation of extract from the polymer due to the inability of absorption of PLA in the region from 280 to 750 nm.

### Kinetics of extracts release from sterecomplexed microparticles in vitro

To determine the release of *S. cadmica* extract from sc-PLAs, 20 mg of MPs in 1 mL of phosphate buffer saline (pH 7.4, 0.1 M) was transferred to the dialyzed membrane (MWCO 8000 Da), and placed into the vial filled with 3 mL of phosphate buffer solution. At the selected time interval, the 1.5 mL of release medium was withdrawn and replaced with fresh media and maintained at 37 °C. The amount of released extracts was quantified by measurement of fluorescence intensity (λ_ex_ = 324 nm, λ_em_ = 431 nm) on a Fluorog-3 fluorimeter (Horiba Jobin–Yvon, Kyoto, Japan). The measurement was repeated three times, and the final results were the average data. The release experiment was also conducted in acetic buffer (0.1 M, pH 5.0) and Tris buffer (0.1 M, pH 8.7).

### Characterization

^1^H NMR spectra were recorded in chloroform-d1 on an Avance Neo 400 NMR spectrometer (400.15 MHz for ^1^H, 9.4 T). The number average molecular weights of polymers were determined by gel permeation chromatography (GPC) using an Agilent Pump 1100 Series (preceded by an Agilent G1379A Degasser), equipped with a set of two PLGel 5 μm MIXED-C columns. A Wyatt Optilab Rex differential refractometer (RI), and a Dawn Eos (Wyatt Technology Corporation, Sanata Barbara, USA) laser photometer (MALLS) were used as detectors. Dichloromethane (DCM) was used as an eluent at a flow rate of 0.8 mL/min at room temperature. Differential scanning calorimetry (DSC) analyses were performed under a nitrogen atmosphere at heating and cooling rates of 10 °C/min, on a DSC 2500 Discovery, TA Instrument. The measurements were performed from − 0 to 240 °C. The temperature and heat flows were both calibrated with indium. Thermogravimetric analyses (TGA) were performed by placing approximately 5 mg of polymer sample in a measuring cell. Measurements were registered under nitrogen flow by heating the samples from ambient temperature to 600 °C at a heating rate of 20 °C min^−1^ on a Hi-Res TGA 2950 thermogravimetric analyser (TA Instruments, New Castle, USA).

### In vitro biocompatibility

#### Cell cultures and stimulation conditions

The L929 mouse fibroblasts (purchased in LGC Standards, Middlesex, UK), *Cavia porcellus* (guinea pig) primary gastric epithelial cells isolated from gastric tissue, guinea pig fibroblasts CRL-1405 ATTC (purchased in American Type Culture Collection, Rockville, Manassas, VA, USA), and human THP-1 monocytes (purchased in ATCC TIB-202) or modified THP1-X Blue cells (purchased in Invitrogen, San Diego, CA, USA), were used in this study. Cells were cultured and passaged as previously described^16–18,76^.

Human THP-1 cells were grown in Roswell Park Memorial Institute (RPMI-1640) medium supplemented with 10% heat-inactivated fetal calf serum (FCS), 100 U/mL penicillin, 100 U/mL streptomycin, 2 mM/mL l-glutamine at 37 °C, (all in Biowest, Nuaillé, France), in a humid atmosphere containing 5% CO_2_.

THP1-X Blue cells (Invitrogen, San Diego, CA, USA) were maintained in complete RPMI-1640 (cRPMI-1640) medium (Sigma Aldrich, Saint Louis, MO, USA), containing 10% FCS, with antibiotics: penicillin (100 U/mL), streptomycin (100 μg/mL) (all in Biowest, Nuaillé, France), and selective agents: normocin (100 µg/mL) and blastocidin (10 µg/mL) (Invitrogen, San Diego, CA, USA), at 37 ℃ in a humidified atmosphere of a cell culture incubator.

THP-1XBlue cells were obtained by transfection of THP-1 cells with a reporter plasmid, the expression of which leads to the secretion of alkaline phosphatase (SEAP) under the control of a promoter induced by the transcription factors nuclear factor kappa B (NF-κB) and activator protein 1 (AP-1). Upon stimulation of the cell surface toll-like receptor (TLR), transcription factors are activated and cells secrete SEAP, which can be detected using the commercial QUANTI-Blue reagent (Invitrogen, San Diego, CA, USA).

All cells were stimulated for 24 h with *S. cadmica* extracts alone (root- SCRE or aerial part-SCAPE) or with stereocomplexed PLA microparticles: sc-PLA (lmm); sc-PLA (mmm) unloaded or loaded with *S. cadmica* extracts: sc-PLA-SCAPE (lmm); sc-PLA- SCAPE (mmm); sc-PLA-SCRE (lmm); sc-PLA-SCRE (mmm), in non-toxic concentrations determined experimentally (2.5 mg/mL)^74^, or as a positive control standard *Escherichia coli* LPS (serotype O55:B5, Sigma Aldrich, St. Louis, Missouri, USA) at a concentration of 1 µg/mL. Four independent experiments were carried out in triplicate for each experimental variant.

#### Cell viability assay

Biocompatibility of *S. cadmica* extracts (root and aerial part, separately) and stereocomplexed PLA microparticles, empty or loaded with extracts, as described above, was tested using the (3-(4,5-dimethylthiazol-2-yl)-2,5-diphenyltetrazolium bromide) (MTT) reduction assay according to the ISO norm 10993-5 (Biological evaluation of medical devices-Part 5: Tests for in vitro cytotoxicity; International Organization for Standardization, 2009) as previously described^74^. For this purpose we used standard L929 mouse fibroblasts, guinea pig primary gastric epithelial cells or fibroblasts, as well as human THP1 monocytes. MTT reduction relative to untreated cells (%) = (absorbance of treated cells/absorbance of untreated cells × 100%) × 100%. Four independent experiments were carried out in triplicate for each experimental variant.

#### DNA damage

Soluble *S. cadmica* extracts, stereocomplexed PLA loaded with *S. cadmica* extracts (sc-PLA) or *E. coli* LPS (as a positive control) were added to the cultures of guinea pig primary gastric epithelial cells or fibroblasts for 24 h. DNA damage was determined using the HCS DNA Damage Kit, (Thermo Fisher Scientific, Waltham, MA, USA) as recommended by the manufacturer. DNA damage was identified by antibody against phosphorylated H2AX (Ser139), which is induced in response to double-strand break (DSB) formation. Cell nuclei were stained with 4ʹ,6-diamidyno-2-fenyloindol (DAPI) and visualized under the fluorescence microscope, as previously described^15^. Four independent experiments were performed in triplicate.

#### Monocyte activation assay

The THP-1X Blue cells were placed in 96-well tissue culture plates (5 × 10^4^ cells/well), and incubated for 24 h with *S. cadmica* extract (root and aerial part) alone or sc-PLA under the conditions of incubator. The cells in culture medium alone (cRPMI-1640) served as a negative control, whereas cells treated with *E. coli* LPS O55:B5 (1 μg/mL) were a positive control. At the endpoint of incubation, 20 μL of cell culture supernatants were added to 180 μL of chromogen-substrate solution (Quanti-Blue reagent), and the SEAP activity as a marker of cell activation was assessed after 2 h. The optical density at 620 nm was measured using a multimode microplate reader SpectraMax i3 (Molecular Devices, San Jose, CA, USA). Four independent experiments were carried out in triplicate for each experimental variant.

#### Cell apoptosis

Primary gastric epithelial cells and guinea pig fibroblasts after exposure to *S. cadmica* extract (root and aerial part) alone, sc-PLA or *E. coli* LPS (as a positive control) were immunohistochemically stained for the presence of early pro-apoptotic caspase 3 (CC3) or middle apoptosis stage caspase 9 (CC9) as well as late apoptosis protein carbamoyl-phosphate synthetase 2 asparate transcarbamylase, and dihydroorotase (CAD). The procedure of staining was performed using fluorescently labeled rabbit specific primary antibodies (Santa Cruz Biotechnology, Dallas, TX, USA), and then anti-rabbit Alexa Fluor 488-IgG secondary antibody (Invitrogen, Waltham, MA, USA), as previously described^16–18^**.** The amount of CC9 and CAD was determined by measuring fluorescence intensity, at 495 nm excitation and 519 nm emission, using a multifunctional SpectraMax i3 reader (Molecular Devices, San Jose, CA, USA). Four independent experiments were carried out in triplicate for each experimental variant. Apoptosis was also determined by terminal deoxynucleotidyl transferase dUTP nick end labeling (TUNEL) assay as previously described^16–18^. The cell nuclei were stained using the fluorescent dye DAPI (Sigma-Aldrich, Saint Louis, MO, USA), which has a strong affinity to the AT base pair in DNA, as previously described^14^. Cell nuclei were imaged under a fluorescent microscope (Zeiss, Axio Scope, A1, Jena, Germany), at a wavelength of 358 nm (excitation) and 461 nm (emission), and the percentage of cells with blebbing nuclei was assessed. Four independent experiments were performed in triplicate.

### In vivo biocompatibility of tested microparticles

Biocompatibility of tested microparticles was assessed according to norm EN ISO 10993-1 “Biological evaluation of medical devices/Evaluation and testing in the risk management process/Biological evaluation process/Research in biological evaluation”.

### Ethical statements

All experiments involving animals were developed according to the Animal Research: Reporting of In Vivo Experiments (ARRIVE) guidelines and guidelines and regulations EU directive (Directive 2010/63/EU of the European Parliament and of the Council of 22 September 2010 on the protection of animals used for scientific purposes (Dz.U. L 276 z 20.10.2010, s. 33–79) and were approved by the Local Ethics Committee (LKE9) for Animal Experiments of the Medical University of Lodz, Poland, which was established by the Ministry of Science and Higher Education in Poland (Ethics Committee decision number: ŁB16/234/2022. Both genders of three-month guinea pigs (five animals per group, respecting the 3R principle), free of pathogens, were bred and housed in the Animal House at the Faculty of Biology and Environmental Protection, University of Lodz (Poland), in accordance with Laboratory Animal Resources Commission standards. The animals were kept in air-conditioned rooms at 20–24 °C in cages with free access to drinking water and food pellets ad libitum. They were exposed to a 12 h light/dark cycle.

#### Assessment of local and systemic effects after intradermal injection of tested microparticles or per os inoculation of guinea pigs

The animals were shaved from the dorsal area without anesthesia at the central site of the trunk 24 h before testing, and then 0.2 mL of tested suspension of microparticles in 0.85% NaCl, or diluent alone, were administered subcutaneously. Microparticles: sc-PLA-(mmm), sc-PLA-SCAPE (mmm) and sc-PLA-SCRE (mmm), were incubated with physiological saline for 72 h at 37 °C (according to the norm PN EN ISO 10993-12:2012E). The animals were monitored daily for water and food intake and behavioral symptoms. Skin reactions, defined as erythema and edema, were evaluated after 24, 48 and 72 h according to a skin reaction scoring system^77^. The appearance of edema and erythema was assessed and graded daily according to the Primary Irritation Index (PII) = 0/3, where 0 indicates that the irritation is negligible. After 72 h, the animals were euthanized with an overdose of sodium barbiturate (Morbital, Biowet, Puławy, Poland), and then the blood and organs (spleen, liver) were collected and examined to exclude tissue disorders, and used for further testing.

Before *per os* inoculation, with sc-PLA (24 h), guinea pigs received orally 1 mL of 0.2N NaHCO_3_ without anesthesia to increase the stomach pH to neutral, and after 5 min 1 mL of tested microparticles: sc-PLA (mmm), sc-PLA-SCAPE (mmm) and sc-PLA-SCRE (mmm) in 0.85% NaCl. Control animals received 1 mL of 0.85% NaCl*.* Nonpolar microparticles were prepared according to the norm PN EN ISO 10993-12:2012E. After 24 h, 48 and 72 h: (from inoculation with microparticles, the animals were euthanized by administering a lethal dose (overdosage, 100 mg/kg.b.m) of sodium barbiturate (Morbital, Biowet, Puławy, Poland), and then the blood and organs (spleen, liver) were collected and examined to exclude tissue disorders, and used for further testing.

In all animals the proliferative activity of spleen leucocytes was determined towards cells stimulated with phytohemagglutinin (PHA) (Sigma-Aldrich, Saint Louis, MO, USA) as positive control or cells in culture medium alone (spontaneous proliferation). Briefly, splenic cell suspensions in RPMI 1640 supplemented with 10% FBS, 2 mM l-glutamine and 100 ug/mL penicillin/streptomycin were added to 96-well plate (5 × 10^5^ cells/well). PHA was added to the final concentration of 5 µg/mL. After 72 h cultivation, the culture medium was removed, and cells were frozen at − 70 °C until further processing. Proliferation assessment was performed on the basis of incorporation of radioactive thymidine—^3^H[dRT] to DNA of dividing cells, as previously described^16^. The concentration of alanine aminotransferase (ALT) and aspartate aminotransferase (AST) in liver homogenates and serum samples was determined by the commercial ELISA (MyBiosoure, San Diego, USA), with the sensitivity 0.06 ng/mL and < 0.118 ng/mL, respectively, according to the attached protocol. Furthermore, the level of serum pro-inflammatory cytokines: tumor necrosis factor alfa (TNF-α) and interleukin (IL)-1B was examined by the ELISA (Thermo Fisher Scientific, Waltham, MA, USA), a sensitivity 1.7 pg/mL (TNF α) and 1 pg/mL (IL-1B), respectively, as recommended by the manufacturer. Three independent experiments were performed in triplicate for each experimental variant.

### Isolation and stimulation of guinea pig bone marrow macrophages

The guinea pig bone marrow macrophages were isolated to cRPMI medium from tibias and femurs as previously described^78^. Cells were adjusted to the density of 5 × 10^6^ cells/mL in cRPMI culture medium and underwent stimulation with *H. pylori* or *S. cadmica* extracts*. H. pylori* reference strain CCUG 17874 (Culture Collection, University of Gothenburg, Gothenburg, Sweden), positive for vacuolating cytotoxin A (VacA) and cytotoxin associated gene A (CagA) protein, was cultured under microaerophilic conditions according to the previously described procedure^16^. Macrophages were stimulated with *H. pylori* using the multiplicity of infection (MOI): 10:1 while *S. cadmica* extracts were used at a concentration 1.25 mg/mL. The procedure of macrophage stimulation was as follows: priming with *S. cadmica* extract for 24 h and 5 days restimulation with the homologous *S. cadmica* extract; priming with *S. cadmica* extract for 24 h, 5 days restimulation with *H. pylori*; priming with *S. cadmica* extract for 24 h, 5 days restimulation with *H. pylori,* and additional 24 h restimulation with homologous *S. cadmica* extract. Stimulated or unstimulated control cells were then examined for phagocytic activity in conjunction with an expression of CD11b activation marker and production of cytokines, TNF-α and IL-10.

#### Phagocytosis

Bone marrow macrophages (5 × 10^6^ cells/mL) were applied to the wells of a 96-well plate (100 µL/well), and stimulated with S*. cadmica* extract (aerial part or root) or with live *H. pylori* as described above. Phagocytosis was assessed using fluorescein-labeled *Escherichia coli*, as recommended by the manufacturer (Vybrant Phagocytosis Assay Kit, Thermo Fisher Scientific, Waltham, MA, USA). Intensity of phagocytosis was determined by measuring the fluorescence using a multifunctional reader SpectraMax i3 (Molecular Devicesat, San Jose, CA, USA) at 495 nm (excitation) and 515 nm (emission). Four independent experiments were carried out in triplicate for each experimental variant.

#### Surface deposition of CD11b: immunofluorescence

The guinea pig bone marrow macrophages after priming and restimulation as described above, were prepared for staining with fluorescently labeled primary and secondary antibodies as previously described^16^. We used rabbit anti-CD11b antibodies (Thermo Fisher Scientific, Waltham, MA, USA), diluted 1:200 in 1% bovine serum albumin (BSA) in phosphate buffered saline (PBS). Cells were then treated with the secondary goat anti-rabbit antibody Alexa Fluor488-labeled (Invitrogen, CA USA), diluted 1:200. The intensity of fluorescence was measured using a multifunctional reader SpectraMax i3 (Molecular Devicesat, San Jose, CA, USA) at the wavelengths for Alexa Fluor488 (excitation 495 nm, emission 519 nm). Four independent experiments were carried out in triplicate for each experimental variant.

#### ELISA for TNF-α and IL-10

After stimulation of macrophages, cell culture supernatants were tested for TNF-α and IL-10 by the commercial ELISA (Thermo Fisher Scientific, Waltham, MA, USA), with the sensitivity 1.7 pg/mL (TNF-α) and 1 pg/mL (IL-10) on the basis of standard curves for these cytokines, as recommended by the manufacturer. Four independent experiments were carried out in triplicate for each experimental variant.

### Statistical analysis

Data were expressed as median values ± range. The differences between groups were tested using the non-parametric Mann–Whitney *U* test or Kruskal–Wallis test. For statistical analysis the Statistica 13 PL software (https://statistica.software.informer.com/13.3software) (Kraków, Poland) was used. Results were considered statistically significant when *p* < 0.05.

## Supplementary Information

Supplementary Information.

## Data Availability

All data generated or analyzed during this study are included in this published article or Supplementary File.
