# Supplementary material for: Stereocomplexed microparticles loaded with Salvia cadmica Boiss. extracts for enhancement of immune response towards Helicobacter pylori
Source: Sci Rep. 2023 Apr 29;13:7039. doi: 10.1038/s41598-023-34321-6 (PMC10148839; doi:10.1038/s41598-023-34321-6)
Supplement: Supplementary file 1 — Supplementary Information. [file 41598_2023_34321_MOESM1_ESM.docx]

Supplementary Information

**Stereocomplexed microparticles loaded with *Salvia cadmica* extracts for enhancement of immune response towards *Helicobacter pylori***

Weronika Gonciarz, Magdalena Chmiela, Bartłomiej Kost, Ewelina Piątczak, and Marek Brzeziński

**Table of Contents**

**Figure S1.** ^1^H NMR of used PLAs for MPs preparation with low molar mass.

**Figure S2.** ^1^H NMR of used PLAs for MPs preparation with medium molar mass.

**Figure S3.** SEC chromatograms of PLA with low molar mass and PLA with medium molar mass.

**Figure S4.** DSC thermograms of blank and extracts-loaded microparticles composed of low molecular mass PLAs.

**Figure S5.** DSC thermograms of blank and extracts-loaded microparticles composed of medium molecular mass PLAs.

**Figure S6.** TGA curves of blank and extracts-loaded stereocomplexed microparticles.

**Figure S7.** Exemplary FTIR spectra of stereocomplexed microparticles showing the presence of a band at 909 cm^-1^.

**Figure S8.** The calibration curve used for the determination of encapsulation efficiency

**Table S1.** Thermal parameters of obtained stereocomplexed microparticles (I and II heating run), before and after loading of the extracts.

**Table S2.** The decomposition temperature values for obtained blank and microparticles loaded with extracts.


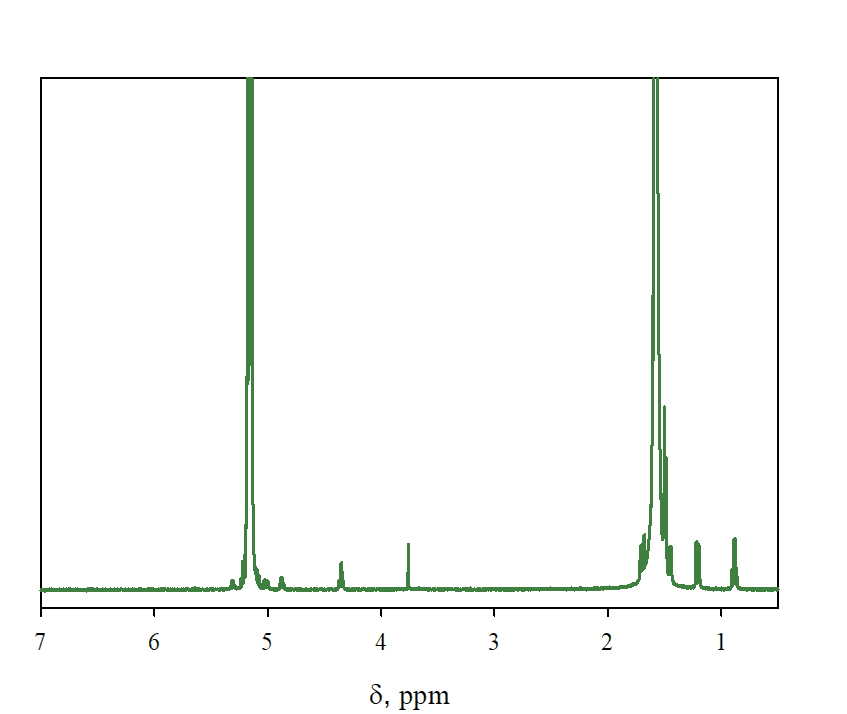


Figure S1. ^1^H NMR of used PLAs for MPs preparation with low molar mass.

PLA - poly(lactic acid), MPs - microparticles


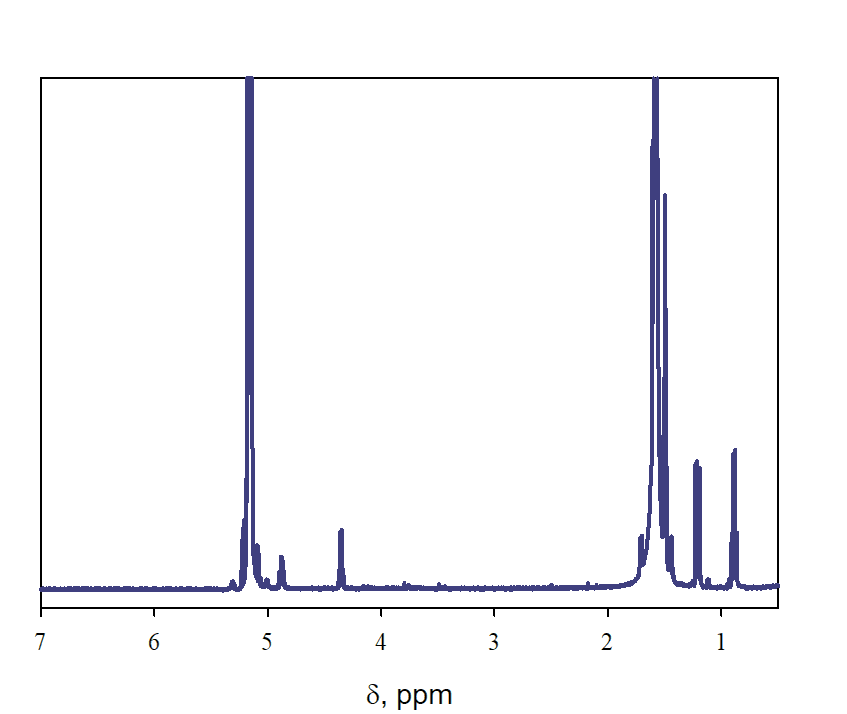


Figure S2. ^1^H NMR of used PLAs for MPs preparation with medium molar mass.

PLA - poly(lactic acid), MPs - microparticles


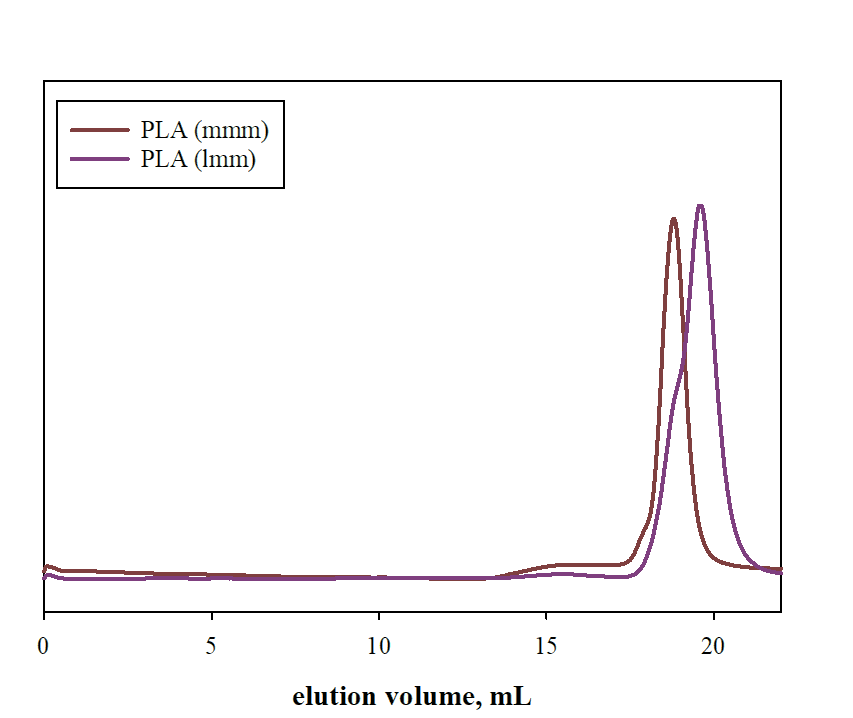


Figure S3. SEC chromatograms of PLA with low molar mass and PLA with medium molar mass. PLA - poly(lactic acid), mmm – medium molecular mass, lmm – low molecular mass


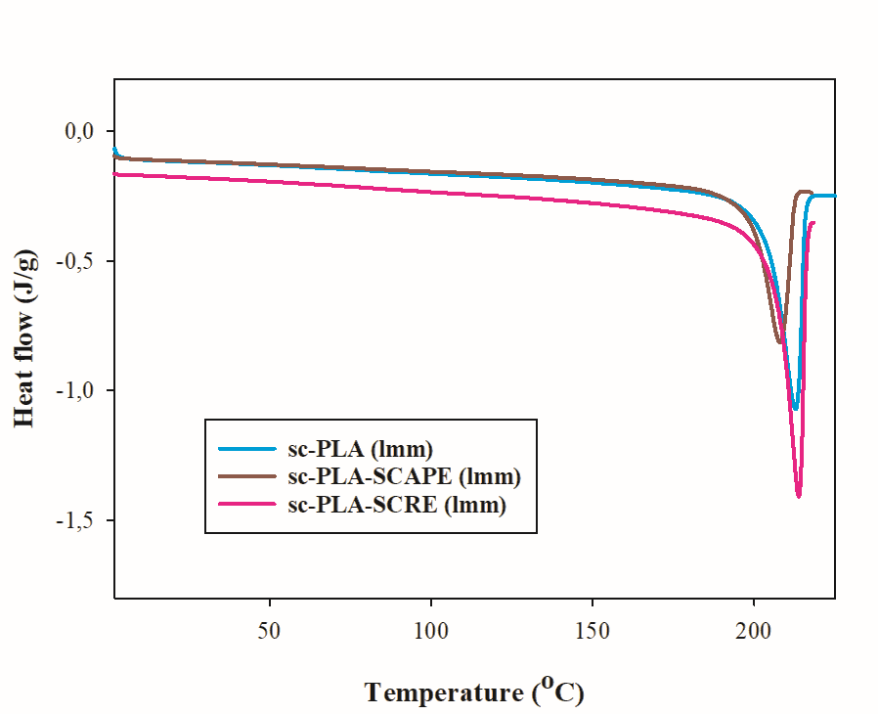


Figure S4. DSC thermograms of blank and extracts-loaded microparticles composed of low molecular mass PLAs showing the formation of stereocomplex crystallites since their melting is above 200 °C (II heating run). DSC - differential scanning calorimetry, sc-PLA (lmm) - stereocomplexed microparticles (sc-PLA) of low molecular mass, SCAPE – *S. cadmica* aerial part extract, SCRE – *S. cadmica* root extract.


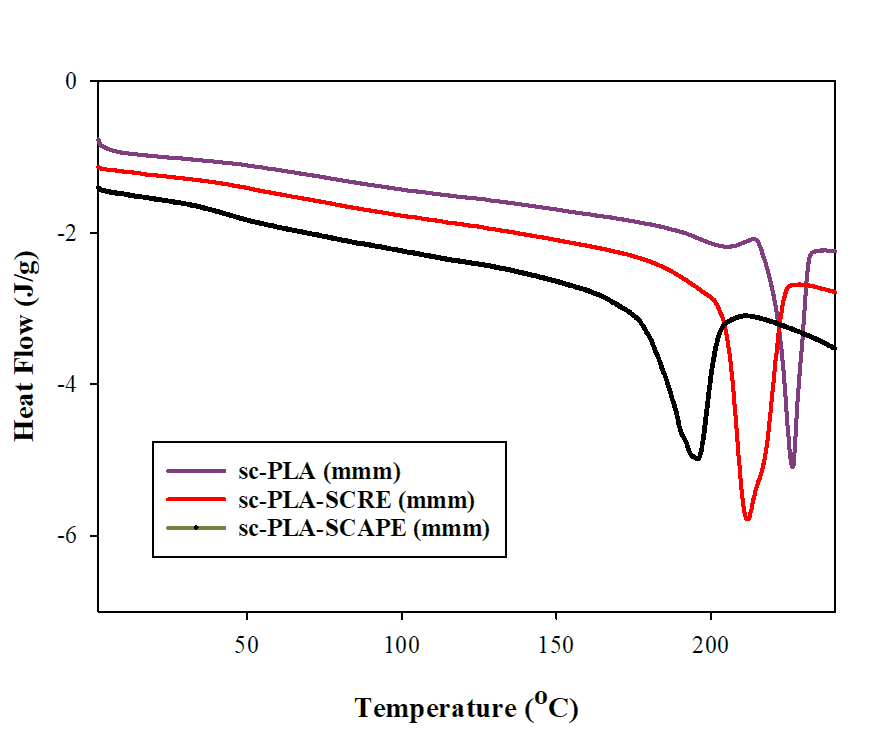


Figure S5. DSC thermograms of blank and extracts-loaded microparticles composed of medium molecular mass PLAs showing the formation of stereocomplex crystallites since their melting is above 190 °C. DSC - differential scanning calorimetry, sc-PLA (mmm) - stereocomplexed microparticles of medium molecular mass, SCARE – *S. cadmica* aerial part extract, SCAPE – *S. cadmica* root extract.


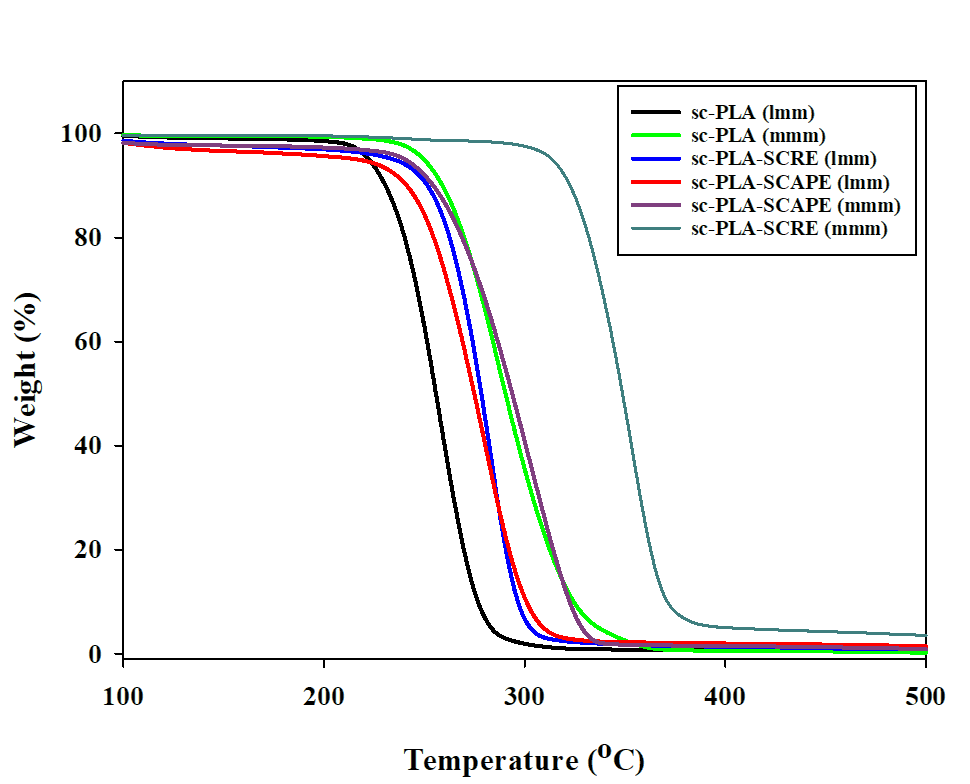


Figure S6. TGA curves of blank and extracts-loaded stereocomplexed microparticles. TGA- thermogravimetric analyses, sc-PLA (Imm) – stereocomplexed microparticles of low molecular mass, sc-PLA (mmm) - stereocomplexed microparticles of medium molecular mass, SCARE – *S. cadmica* aerial part extract, SCAPE – *S. cadmica* root extract.


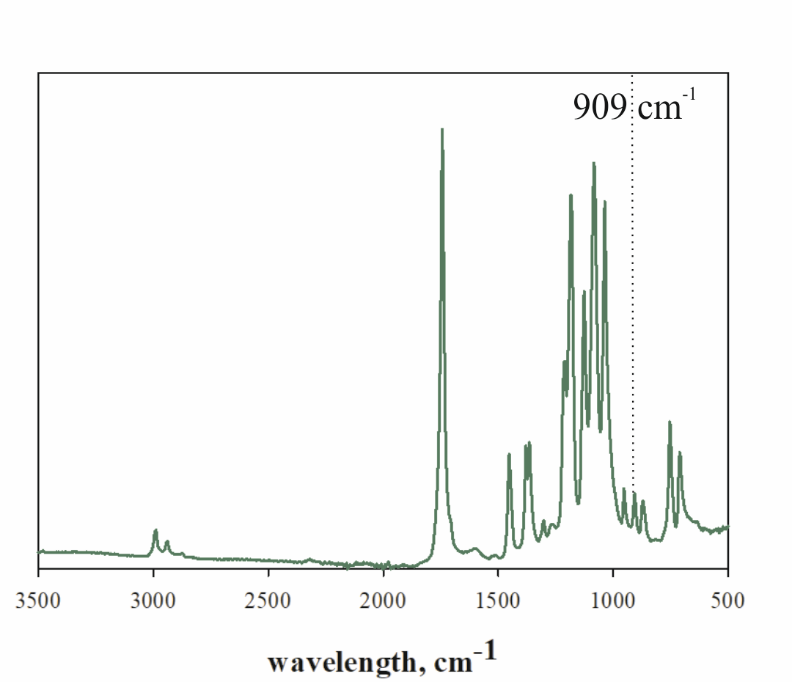


Figure S7. Exemplary FTIR spectra of sterecomplexed microparticles of medium molecular mass loaded with *S. cadmica* aerial part extract (sc-PLA-SCAPE (mmm)) showing the presence of a band at 909 cm^-1^. FTIR - Fourier transform infrared spectroscopy.

Figure S8. The calibration curve used for the determination of encapsulation efficiency. The measurement was done at 324 nm.

Table S1. Thermal parameters of obtained stereocomplexed microparticles (I and II heating run), before and after loading of the extracts.

| **Sample** | ***T*_m_**  **I heating**  **[°C]** | ***ΔH_m_***  **I heating**  **[J/g]** | ***T*_m_**  **II heating**  **[°C]** | ***ΔH_m_***  **II heating**  **[J/g]** |
| --- | --- | --- | --- | --- |
| sc-PLA (lmm) | 212 | 105 | 213 | 113 |
| sc-PLA-SCAPE (lmm) | 211  159 | 102  7 | 208 | 95 |
| sc-PLA-SCRE (lmm) | 212 | 108 | 214 | 104 |
| sc-PLA (mmm) | 231 | 110 | 226 | 82 |
| sc-PLA- SCAPE (mmm) | 232 | 99 | 195 | 48 |
| sc-PLA- SCRE (mmm) | 233 | 75 | 211 | 62 |

Sterocomplexed microparticles of low molecular mass - sc-PLA (lmm) or medium molecular mass - sc-PLA (mmm) loaded with *S. cadmica* aerial part extract (SCAPE) or *S. cadmica* root extract (SCRE).

Table S2. The decomposition temperature values for obtained blank and microparticles loaded with extracts.

| sc-PLA (lmm) | sc-PLA-SCAPE (lmm) | sc-PLA-SCRE (lmm) | sc-PLA (mmm) | sc-PLA- SCAPE (mmm) | sc-PLA- SCRE (mmm) |
| --- | --- | --- | --- | --- | --- |
| **257 °C** | **280 °C** | **283 °C** | **288 °C** | **302 °C** | **353 °C** |

Sterocomplexed microparticles of low molecular mass - sc-PLA (lmm) or medium molecular mass - sc-PLA (mmm) loaded with *S. cadmica* aerial part extract (SCAPE) or *S. cadmica* root extract (SCRE).
